# The Importance of Comparing New Technologies (AI) to Existing Tools for Patient Education on Common Dermatologic Conditions: A Commentary

**DOI:** 10.2196/71768

**Published:** 2025-04-01

**Authors:** Parker Juels

**Affiliations:** 1 School of Medicine, University of Colorado Anschutz Medical Campus, 13001 E 17th Pl, Aurora, 80045, United States, 1 3032499779

**Keywords:** artificial intelligence, ChatGPT, atopic dermatitis, acne vulgaris, actinic keratosis, rosacea, AI, diagnosis, treatment, prognosis, dermatological diagnoses, chatbots, patients, dermatologist

In their study, Chau and colleagues discussed the sufficiency of multiple artificial intelligence resources in answering possible patient questions on common dermatological conditions [1]. It is very important to examine the reliability of artificial intelligence, especially as it relates to patient care and is becoming increasingly widespread. It is also very beneficial to have a comparison of what artificial intelligence is available and what their unique weaknesses are. However, we do have a plethora of existing resources, including paper handouts, peer-reviewed journals, patient-centered websites, and physical media, all of which have been providing reliable information to patients for many years. Because artificial intelligence is not a harmless technology [2,3], the proven efficacy of the existing resources [4,5], and the reported errors in artificial intelligence answers [1], it is not sufficient to only prove that artificial intelligence could be reliable but also prove that it has advantages compared to existing tools.

Research has demonstrated moderate improvement in patient care with either written or online information provided by healthcare providers [4]. Additionally, more recent research has shown that information provided through patient portals improves patient understanding and healthcare outcomes [5]. Since there are established benefits of providing information directly to patients and the existence of a plethora of reliable websites that provide quality information, it is important to compare any new intervention, including artificial intelligence, to these existing information forms.

It is especially important to establish a significant benefit of artificial intelligence compared to our existing resources due to the detrimental outcomes that increased use could have on the environment and patient knowledge. Artificial intelligence usage is an energy-demanding and resource-consuming practice that requires an outsized water consumption and carbon output compared with traditional search inquiries [2,3]. Additionally, the study mentions that there have been reported cases of artificial intelligence making up sources or providing completely inaccurate information. This is something the study did examine, finding no evidence of hallucinations/fabrications. However, it is important to have a better understanding of how likely these events are on a larger scale and how to prevent them before recommending patient use of artificial intelligence.

Without true controls, the study’s conclusions do not provide adequate confidence in recommending patient usage of artificial intelligence. There should also be further consideration of how AI can be used to augment, not replace existing forms of patient education. Further research that considers the advantages of existing resources and pitfalls of artificial intelligence is needed before widespread artificial intelligence use in patient care.
